# HIV is still a major public health problem among pregnant women attending ANC in Referral Hospitals of the Amhara Regional State, Ethiopia: a cross sectional study

**DOI:** 10.1186/s12905-022-02059-4

**Published:** 2022-11-24

**Authors:** Workie Zemene Worku, Telake Azale, Tadesse Awoke Ayele, Dawit Kassahun Mekonnen

**Affiliations:** 1 Department of Community Health Nursing, School of Nursing, College of Medicine and Health Sciences, University of Gondar, Gondar, Ethiopia; 2 Department of Health Education and Behavioral Sciences, Institute of Public Health, College of Medicine and Health Sciences, University of Gondar, Gondar, Ethiopia; 3 Department of Epidemiology and Biostatistics, Institute of Public Health, College of Medicine and Health Sciences, University of Gondar, Gondar, Ethiopia; 4 Department of Gynaecology and Obstetrics, School of Medicine, College of Medicine and Health Sciences, University of Gondar, Gondar, Ethiopia

**Keywords:** HIV infection, Pregnant women, Determinant factors, Amhara Regional State, Ethiopia

## Abstract

**Background:**

The burden of HIV is disproportionately higher among women of reproductive age contributing more than half of the global share. The situation in Ethiopia is not exceptional. The present study was done to determine the proportion of HIV among pregnant women in Amhara Regional State, Ethiopia.

**Method:**

Institutions-based cross-sectional study was conducted from October 2020 to December 2020. Systematic random sampling technique was used to select 538 study participants from pregnant women who had ANC follow-up in Referral Hospitals of the Amhara Regional State. Data on socio-demographic, clinical, obstetric, behavioral as well as psychosocial characteristics were gathered using an interviewer administered structured and standardized instruments. The data was entered into Epi-Data Manager V4.6.0.0 and exported to STATA version 14 for data analyses. Descriptive statics were computed to summarize the participant’s characteristics. Bi-variable and multivariable logistic regression analyses were conducted to identify the association between dependent and independent variables. Independent variables with a *p*-value of less than 0.05 were considered to be statistically significant at 95% confidence level (CI).

**Results:**

The proportion of HIV infection among pregnant women was 8.68% (95% CI: 6.5, 11.4). Completing secondary school education (Adjusted Odds Ratio (AOR = 0.15; 95% CI: 0.04—0.53), graduated from college (AOR = 0.03; 95% CI: 0.01—0.22), and family monthly income greater than 8001 ETB (1 USD = 56 ETB) (AOR = 0.19; 95% CI: 0.04—0.87) were protective factors associated with maternal HIV. On the other hand, history of previous abortion (AOR = 7.73; 95% CI: 3.33—17.95) and positive syphilis status (AOR = 10.28; 95% CI: 2.80—37.62) were risk factors associated with maternal HIV status.

**Conclusion:**

The proportion of HIV infection among pregnant women was found to be high. Advanced level of education, relatively higher monthly income, history of abortion and previous syphilis status were associated factors with HIV status. Strengthening women's formal education; empowering women in all spheres of life (especially improving their economic standing that prevents women from engaging in risky sexual practices); educating women about HIV transmission methods and HIV prevention and control strategies using behavior change intervention strategy prepared for women to reduce their vulnerability; advocating for the use of family planning to reduce unsafe abortions and syphilis; as well as regular screening and testing for syphilis are recommended.

## Plain language

Millions lost their lives for HIV/AIDS while many more live with the virus with significantly compromised degree of quality of life. Women are more affected than men because of various contributing factors. This study was aimed to determine the proportion and associated factors of HIV status among pregnant women attending ANC at referral hospitals of the Amhara Regional State, Ethiopia. The study was conducted from October 2020 to December 2020. A total of 538 pregnant women were included in the study selected from three referral hospitals.  An interviewer-administered questionnaire was used to collect the data. A binary logistic regression analysis was used to identify the association between factors of the study participants and HIV status. The result revealed that the overall proportion of HIV among pregnant women was 8.68%. Women who completed secondary education, graduated from higher education and having a relatively adequate monthly income were found to have less chance of contracting HIV. On the other hand, pregnant women who had history of previous abortion and positive for syphilis had a higher chance to be infected with HIV. Such a high HIV proportion among the participants in the study area implies that there might be lack of awareness about HIV infection transmission methods as well as HIV prevention and control strategies. In this connection, behavior change intervention strategy prepared specifically for women is recommended to reduce their vulnerability. Besides promoting the use of family planning methods to reduce unsafe abortions and syphilis as well as regular screening and testing for syphilis are important to consider.

## Introduction

It is over three decades now that HIV/AIDS has emerged as a global epidemic. Since its onset in the early 1980s, an estimated 79.3 million people got infected with HIV; 36.3 million people died; a plethora of people have been in pain, grief, and living with chronic disease worldwide [1–3]. In 2020, an estimated 37.7 million people were living with HIV globally while a total of 1.5 million people acquired the virus. Of these, women accounted for 50% [1, 4]. Sub-Saharan Africa (SSA) remains among the regions hit hard by the infection contributing for nearly two-thirds of the global total HIV cases [5, 6].

Ethiopia is one of the top 25 countries globally with new HIV infections [7]. According to the Joint United Nations Program on HIV/AIDS (UNAIDS) 2020 country fact sheet, Ethiopia has a total of 620,000 people living with HIV. Of these, 360,000 were women of childbearing age with 8,900 adults newly infected [8]. Despite Ethiopia's remarkable progress in preventing and controlling the epidemic, HIV continues to be a major public health issue among Ethiopia's reproductive and working populations [7, 9].

Specific to pregnant women, an estimated 1.3 million pregnant women worldwide were living with HIV, with 90% of those living in SSA nations in 2017 [6]. A meta–analysis study in SAA showed that the pooled incidence of HIV among pregnant women was 2.1% [10]. In Ethiopia, the 2014 national antenatal care (ANC) sentinel surveillance showed that the national HIV prevalence among pregnant women was 2.2% with huge regional variations. The top three regional states of Ethiopia with higher prevalence of HIV being Gambela (7.5%), Harari (6.6%), and Amhara (6.1%) [11].

HIV infection raises the risk of maternal morbidity and mortality during pregnancy. Among Sub-Saharan Africa, HIV/AIDS and related illnesses are the major cause of death in women of reproductive age accounting for one-quarter of all deaths during pregnancy and the six-week postpartum period. Moreover, HIV infection among mothers during pregnancy is linked to a variety of negative pregnancy and delivery outcomes including abortion, stillbirth, low birth weight, and premature birth [6, 12–14] HIV can also be transmitted from the infected mother to the baby during pregnancy, childbirth and breastfeeding with a risk of 15% to 45% [14]. The risk of MTCT of HIV ranges from 25 to 48% in developing countries which is higher than the case in developed nations [15].

However, antiretroviral treatment and other interventions can reduce such a risk to below 5% [14]. This can be achieved via initiating early Antiretroviral Treatment (ART) as per the guideline, plan the mode of delivery as well as provide other necessary interventions through pregnancy, childbirth and breastfeeding [16]. Increasing access to PMTCT is a vital component of the global strategies to achieve the SDG-3 which is ending HIV/AIDS by 2030 through reduction of new HIV infection among paediatric age groups [17, 18].

Various studies show that a range of factors are associated with women’s HIV seropositive status. Low maternal educational status [19, 20] history of previous abortion [21], and positive for syphilis test [22] were risk factors for HIV positive sero status among pregnant women.

In spite of its enormous burden among pregnant women, there is a dearth of studies in Amhara Regional State, where the current study was conducted. In addition to determining the proportion of HIV infection among pregnant women, our study made an effort to include the most important but overlooked predictor variables, which most previous studies did not address, such as psychosocial variables together with other predictor variables.

Determining the proportion of HIV among pregnant women can be considered as a good proxy to estimate HIV among the general population. The present study provides up to date evidence on the current burden of HIV among pregnant women destined to serve as an input to monitor the course of HIV epidemics, and the progress of four-pronged approach adopted by the United Nations (UN) to the prevention of mother-to-child transmission of HIV (PMTCT), allocate resources to strengthen prevention and control measures as well as policy considerations. It also helps to measure country progress towards achieving the sustainable development goal (SDG-3) in the time of the COVID-19 pandemic since reports showed that COVID-19 has an impact on the journey to end HIV in 2030 [23, 24].

This study was, therefore, conducted with the aim of determining the proportion and identifying factors associated with HIV infection among pregnant women attending ANC clinics at referral Hospitals of The Amhara Regional State, Ethiopia.

## Methods

### Study design and setting

An institution-based cross-sectional study was conducted from October 2020 to December 2020 to determine the proportion of HIV and its associated factors among pregnant women attending ANC at referral hospitals of the Amhara Regional State, Ethiopia. There were six referral hospitals in the region serving 3.5 to 5 million people [25]. In this study, three hospitals (University of Gondar Comprehensive Specialized Hospital, Felege Hiwot Comprehensive Specialized Hospital, and Debre Tabor Referral Hospital) were selected as the study sites out of the six referral hospitals. University of Gondar Comprehensive Specialized Hospital is located in Gondar town, Central Gondar Zone, North West Ethiopia, 727 km away from Addis Ababa [26]. Felege Hiwot Comprehensive Specialized Hospital is located in Bahir Dar; the capital city of the Amhara Regional State. And Debre Tabor Referral Hospital is found in Debre Tabor town, 665 km away from Addis Ababa. All Referral Hospitals of the Amhara Regional State offer focused ANC services, and have a separate ART as well as PMTCT clinic. The Ethiopian government began implementing Option B + (initiation of antiretroviral medication for all expectant mothers) in 2013. Since then, the service has been made available in all health facilities at no cost.

### Source and study population

Pregnant women attending ANC services in the referral hospitals of the Amhara Regional State were the source population. The study population was pregnant women attending ANC services during the study period in the selected referral hospitals of the region.

### Sample size determination

Sample size was determined using single population proportion formula [*n* = (Za/2)^2^ p (1 − p)/d^2^], considering, 6.1% of HIV prevalence among pregnant women in the Amhara Regional State from the 2014 antenatal sentinel surveillance report of Ethiopia [11], 95% level of confidence, 3% margin of error, design effect of 2, and 10% non-response rate. The final sample size was 538.

### Sampling technique and procedure

The study participants were drawn from the three selected referral hospitals of the Amhara Regional State after proportional allocation was done. We assigned the sample size for each of the hospitals based on the number of pregnant women attending ANC in those referral hospitals. The study participants were chosen based on daily flow records of pregnant women seeking ANC at these hospitals. The average daily attendance at the ANC clinics were 30, 20 and 15 in University of Gondar Comprehensive Specialized Hospital, Felege Hiwot Comprehensive Specialized Hospital, and Debre Tabor Referral Hospital, respectively. During the study period, about 1600, 1200 and 900 pregnant women attended ANC at University of Gondar Comprehensive Specialized Hospital, Felege Hiwot Comprehensive Specialized Hospital, and Debre Tabor Referral Hospital, respectively. Consequently, we included 233 from University of Gondar Comprehensive Specialized Hospital, 174 from Felege Hiwot Comprehensive Specialized Hospital, and 131 from Debre Tabor Referral Hospital from a total of 3,700 participants.

Systematic random sampling technique was employed to select the individual study participant. Sampling interval (k) was calculated as k = 3700/538 = 7. Based on this sampling interval, study participants were selected at seven intervals until the required sample was attained.

### Data collection procedures and quality assurance

The data was collected using interviewer-administered, structured as well as standardized questionnaires. The tool consists of items on socio-demographic, obstetric, medical, and behavioral conditions of the study participants. Besides, some clinical data were collected from the charts of the participants. The tool was first developed in English and then translated to the local language, Amharic, which is the participants’ mother tongue, to avoid difficulty in communication and finally translated back to English to verify consistency. In addition, face and content validity were checked and found valid. Specifically, food insecurity was measured using the three-item household hunger score with 3-point Likert scale with Cronbach’s Alpha of 0.74 in the present study. Social support was measured using the Maternity Social Support Scale (MSSS) developed by Webster et al. 2000, with Cronbach’s Alpha of 0.55 in the current study**.** The data were collected by three BSc nurses working in the three referral hospitals and supervised by three MSc Nurses.

To maintain the quality of the data different measures were taken. Firstly, face and content validity of the tool were performed. A range of experts such as gynaecologist, midwives, reproductive health professionals and infectious disease experts participated in the face and content validation process. Accordingly, adjustments were made based on their expertise. Secondly, a pre-test was conducted in Gondar Poly Health Centre among 50 (10%) pregnant women to check clarity and reliability of the tool. Overall, the tool was found to be valid and reliable. Thirdly, training was given to the data collectors and the supervisors on the objective and content of the questionnaire, on how to approach a patient and conduct interviews, as well as on ethical aspects of the study such as how to maintain confidentiality of the information obtained from the research, and how to respect autonomy of the participants. Finally, daily supervision was done throughout the data collection time to maintain the quality of the data.

### Variables of the study

The dependent variable was HIV sero-status of pregnant women. The independent variables include socio-demographic factors (i.e. age of the mother, marital status, maternal educational status, paternal educational status, residency, maternal occupational status, paternal occupational status, family monthly income) obstetric and related factors (i.e. plan of pregnancy, parity, gravidity, gestational age, abortion history, syphilis status), behavioral factors (i.e. alcohol consumption, cigarette smoking) and other factors including level of social support and house hold food security status.

### Operational definitions

HIV positive sero- status: positive HIV antibody test result which is confirmed by a second HIV antibody test, and/or positive virological test [27].

Social support: is a perception of communication of love, caring, trust, or concern of family and friends for an individual. It was measured using the Maternity Social Support Scale (MSSS). The scale contains 6-items with 5-point Likert scale. The total possible score for the scale is 30, and the cut-off points for the scale were set at 0 – 18 (low support), 19 – 24 (medium support), and > 24 (adequate support) [28].

Food security: is defined as a state in which all people at all times have both physical and economic access to sufficient food to meet their dietary needs for a productive and healthy life [29]. In the current study, food insecurity was measured using the three items scale known as Household Hunger Score with 3-point Likert scale. The total possible score for the scale is 6 with cutoff points ranging from 0 – 1 (little to no hunger), 2 – 3 (moderate hunger), and 4 – 6 (severe hunger) [30].

Alcohol use: It was assessed with the question “Have you been drinking alcohol during your current pregnancy?” If the answer was “yes” and took any unit of alcohol during the current pregnancy, then the mother was considered to have alcohol exposure while pregnant.

Cigarette smoking: It was assessed with the question “Have you been smoking since your pregnancy?” If the answer for this question was a “yes” even for once the mother was considered to have a tobacco exposure during pregnancy.

### Data processing and analysis

The data was cleaned, coded and entered in to EpiData Manager V4.6.0.0 and exported to STATA version 14 for recoding and analysis. Descriptive and summary statistics were computed to summarize the characteristics of the participants. The association between HIV sero-status and selected independent variables such as socio-demographic, obstetric, medical and behavioral variables were tested using a binary logistic regression model. Independent variables with *p*-value ≤ 0.2 in the bi-variable analysis were potential candidates for the multivariable logistic regression analysis to control confounders. Variables with *p*-value less than 0.05 in the multivariable binary logistic regression model were considered to be statistically significant at 95% CI. Hosmer and Lemeshow’s goodness of fit test was used to check the model fitness (Prob value = 0.3202).

## Results

### Socio-demographic characteristics of the study participants

A total of 538 pregnant women attending ANC in referral hospitals of the Amhara Regional State during the study period were enrolled in the study. Of these, 530 responses were included in the final analysis with a response rate of 98.5%. The age of the participants ranged from 18—45 years with mean age (± SD) 27.7 (± 5.3) (95% CI: 27.30—28.19). Of the total participants, 468 (88.30%), 519 (97.92%), 459 (86.60%), and 265 (50.00%) were urban residents, married, Orthodox Christian, and housewife by occupation, respectively. One quarter of the participants (25.7%) earned a monthly income less than 2500 ETB (Table 1).

**Table 1:** Socio-demographic characteristics of participants in Referral Hospitals of the Amhara Region, Ethiopia, (*n* = 530)

Characteristics	Number (*n* = 530)	Percent (%)
**Age category**		
< 25 years	185	34.91
26–35	299	56.42
>= 36	46	8.68
Mean age (SD)	530	27.7 (± 5.3)
**Residence**		
Rural	62	11.70
Urban	468	88.30
**Religion**		
Orthodox Christian	459	86.60
Muslim	61	11.51
Catholic/protestant/Adventist	10	1.89
**Marital status**		
Married	519	97.92
Single/widowed/divorced	11	2.08
**Maternal educational status**		
Unable to read and write	88	16.60
Primary school	127	23.96
Secondary school	164	30.94
Higher education	151	28.49
**Husband educational status**		
Do not have husband	11	2.08
Unable to read and write	79	15.22
Primary school	82	15.80
Secondary school	175	33.72
Higher education	183	35.26
**Maternal occupational status**		
Government employee	119	22.45
Private employee	86	16.23
House wife	265	50.00
Students/ farmers/ daily laborer	60	11.32
**Husband occupational status**		
Government employee	165	31.79
Private employee	244	47.01
Farmer	64	12.33
Students/ daily laborer/driver/solider	46	8.86
**Family monthly income**		
< =2500 ETB	136	25.66
2501 to 5000 ETB	174	32.83
5001 to 8000 ETB	95	17.92
> 8001 ETB	125	23.58
Monthly income Mean (± SD)	530	6077 (± 4954.5)

#### Obstetric, medical and related characteristics of the study participants

The result revealed that more than half (59.43%) of the participants were multigravida, and the majority (90.38%) of them have more than three children currently living in the household. Over half (53.02%) of the participants started ANC follow-up in their first trimester. Two hundred forty-six (46.42%) women got screened for HIV in the second trimester of their pregnancy (Table 2).

**Table 2:** Obstetric characteristics of participants in Referral Hospitals of Amhara Region, Ethiopia, (*n* = 530)

Variable	Number (*n* = 530)	Percentage (%)
**Gestational age on screening day**		
First trimester	57	10.75
Second trimester	246	46.42
Third trimester	227	42.83
**Plan of pregnancy**		
Planned	459	86.60
Not planned	71	13.40
**Gravidity**		
Multigravida	315	59.43
Primagravida	215	40.57
**Number of living children**		
<= 3	282	90.38
>= 4	30	9.62
**Month ANC started**		
First trimester	281	53.02
Second trimester	238	44.91
Third trimester	11	2.08
**History of previous abortion**		
No	405	76.42
Yes	125	23.58
**HIV status of the women**		
Negative	484	91.32
Positive	46	8.68
**When HIV status known**		
Before the current pregnancy	39	84.78
During the current pregnancy	7	15.22
**How long living with HIV**		
< =5 year	17	36.96
> 5 year	29	63.04
**Husband tested for HIV**		
Yes	70	13.21
No	460	86.79
**Husband HIV status**		
Positive	32	45.71
Negative	38	54.29
**Tested for syphilis**		
Yes	484	91.32
No	46	8.68
**Syphilis status**		
Yes	19	3.58
No	511	96.42

#### HIV sero-status and related characteristics of the study participants

The overall proportion of HIV positive status among pregnant women was 8.68% (95% CI: 6.5, 11.4). Specifically, it was 9.7% in the participants from University of Gondar Comprehensive Specialized Hospital, 10.4% in Felege Hiwot Comprehensive Specialized Hospital, and 4.6% in Debre Tabor Referral Hospital. Of the total HIV positive women, the majority 39 (84.78%) knew their HIV sero-status before the current pregnancy, and more than half (63.04%) of them were living with HIV for more than five years. On the other hand, 70 (13.21%) pregnant women’s husbands were tested for HIV and of these, 32 (45.71%) were reactive for HIV (Table 2).

#### Obstetric, medical and related characteristics in relation to HIV status

Of the total HIV positive pregnant women, 37 (80.43%) had planned pregnancy; 35 (76.09%) were multigravida; and 16 (34.78%) had history of previous abortion (Table 3).

**Table 3:** Obstetric characteristics by HIV status in Referral hospitals of Amhara region, Ethiopia (*n* = 530)

Variable	HIV sero-status	
	HIV positive n (%)	HIV negative n (%)
**Plan of pregnancy**		
Planned	37 (80.43)	422 (87.19)
Not planned	9 (19.57)	62 (12.81)
**Gravidity**		
Primigravida	11 (23.91)	204 (42.15)
Multigravida	35 (76.09)	280 (57.85)
**History of previous abortion**		
Yes	29 (63.04)	96 (19.83)
No	17 (36.96)	388 (80.17)
**Gestational age on screening day**		
first trimester	6 (13.04)	51 (10.54)
Second trimester	19( 41.30)	227 (46.90)
Third trimester	21 (45.65)	206 (42.56)
**Alcohol consumption during pregnancy**		
Yes	20 (43.48)	235 (48.55)
No	26 (56.52)	249 (51.45)
**Level of Social support**		
low social support	15 (32.61)	155 (32.02)
Medium Support	30 (65.22)	310 (64.05)
Adequate social support	1 (2.17)	19 (3.93)
**Food insecurity**		
Little to no hunger	39 (84.78)	454 (93.80)
Moderate hunger	2 (4.35)	18 (3.72)
Severe hunger	5 (10.87)	12 (2.48)

#### Behavioral characteristics of the study participants

Two hundred fifty-five (48.11%) participants consumed alcohol during pregnancy. Of these, the majority (87.84%) consumed locally prepared alcohol called Tella and 17 (6.67%) revealed to drink more than one type of alcohol. About a two-third (66.27%) of the participants consumed alcohol rarely. On the other hand, none of the pregnant women smoked cigarettes during pregnancy. Three hundred and forty (64.15%) participants received medium social support. A great majority (93.02%) of the participants were reported to have encountered a little to no hunger in the household during pregnancy (Table 4).

**Table 4:** Behavioral characteristics of participants in Referral Hospitals of Amhara region, Ethiopia (*n* = 530)

Variable	Number (*n* = 530)	Percentage (%)
**Alcohol consumption during pregnancy**		
No	275	51.89
Yes	255	48.11
**Type of Alcohol consumed**		
local alcohol or Tella	224	87.84
More than one type of alcohol	17	6.67
other (Beer/Arqi/ Wine)	14	5.49
**Cigarette smoking**		
Yes	0	0
No	530	100
**Frequency of alcohol intake**		
Daily	7	2.75
Once or twice a week	8	3.14
Occasionally	71	27.84
Rarely	169	66.27
**Level of Social support**		
low social support	170	32.08
Medium Support	340	64.15
Adequate social support	20	3.77
Internal consistency (α)		0.5 (moderate)
**Food insecurity**		
Little to no hunger	493	93.02
Moderate hunger	20	3.77
Severe hunger	17	3.21
Internal consistency (α)		0.74(highly reliability)

#### HIV status and associated risk factors

A multivariable binary logistic regression analysis was performed. Consequently, four variables (i.e. maternal educational status, having a history of previous abortion, positive syphilis status, and family monthly income) were found to be statistically significant with maternal HIV status. Women who completed secondary education had an 85% less chance of contracting HIV than those who were illiterate (AOR = 0.15; 95% CI: 0.04—0.53). Similarly, women who graduated from higher education had a 97% less chance to be infected with HIV than women who were illiterate (AOR = 0.03; 95% CI: 0.01—0.22). On the other hand, pregnant women who had a history of previous abortion had almost eight times greater odds to contract HIV infection than those who did not have previous abortion (AOR = 7.73; 95% CI: 3.33—17.95). Besides, women who were positive for syphilis were ten times more likely to be infected with HIV than those women who were negative for syphilis (AOR = 10.28; 95% CI: 2.80—37.62). The result also showed that participants who had a family monthly income of greater than 8,001 ETB were less likely to be infected with HIV by 81% than those who had monthly income of less than 2,500 ETB (AOR = 0.19; 95% CI: 0.04—0.87) (Table 5).

**Table 5:** Bivariable and multivariable analysis of factors among pregnant women in Referral Hospitals (*n* = 530)

Variable	HIV status		COR (95% C.I)	AOR (95% C.I)
	HIV- positive n (%)	HIV-negative n (%)		
**Age**				
<= 25 years	5(10.87)	180(37.19)	1	
26–35	32(69.57)	267(55.17)	4.31(1.65,11.28)	1.89(.57,6.27)
>= 36	9(19.57)	37(7.64)	8.75(2.77,27.63)	3.96(.89,17.44)
**Residence**				
Rural	2 (3.23)	60(96.77)	1	
Urban	44(9.40)	424(90.60)	3.11(.73,13.17)	3.2(.31,33.27)
**Maternal educational status**				
Illiterate	13 (14.77)	75 (85.23)	1	
Primary school	16 (12.60)	111(87.40)	.8316(.37,1.829)	.48(.15,1.53)
Secondary school	11 (6.71)	153 (93.29)	.414(.17,.96)	0.15(.04,.53)*
College/University	6 (3.97)	145(96.03)	.239(.087,.65)	0.03(.01,.22)*
**Husband Educational status**				
Illiterate	4(5.06)	75(94.94)	1	
Primary school	12(14.63)	70(85.37)	3.21(.99,10.43)	2.8(.59,13.11)
Secondary school	14(8.00)	161(92.00)	1.63(.51,5.12)	3.1(.64,15.21)
College/University	13(7.10)	170(92.90)	1.43(.45,4.54)	5.1(.88,29.76)
**Maternal occupational status**				
Government employee	7(5.88)	112(94.12)	1	
Private employee	12(13.95)	74(86.05)	2.59(.97,6.89)	.35(.06,2.11)
House wife	21(7.92)	244(92.08)	1.37(.56,3.33)	.29(.05,1.52)
Student, daily laborer	6(10.00)	54(90.00)	1.77(.57,5.55)	.71(.08,5.85)
**Husband occupation status**				
Government employee	10(6.06)	155(93.94)	1	
Private employee	27(11.07)	217(88.93)	1.92(.90,4.10)	1.8(.58,5.58)
Farmer	2(3.13)	62(96.88)	0.50(.11,2.35)	.14(.01,1.84)
Student, daily laborer	4(8.70)	42(91.30)	1.47(.44,4.94)	.24(.04,1.64)
**Plan of pregnancy**				
Yes	37(8.06)	422(91.94)	.60(.28,1.31)	1.15(.36,3.63)
No	9(12.68)	62(87.32)	1	
**History of Previous abortion**				
Yes	29(23.20)	96(76.80)	6.89(3.64,13.06)	7.73 (3.33,17.95)*
No	17(4.20)	388(95.80)	1	
**Social support**				
low social support	15(8.82)	155(91.18)	1	
Medium Support	30(8.82)	310 (91.18)	1(.52,1.913)	1.63(.66,4.03)
Adequate	1(5.00)	19(95.00)	.543(.07,4.35)	.76(.06,9.47)
**Household food insecurity**				
Little to no hunger	39(7.91)	454(92.09)	1	
Moderate hunger	2(10.00)	18(90.00)	1.29(.28,5.77)	.67(.08,5.39)
Severe hunger	5(29.41)	12(70.59)	4.85(1.63,14.47)	2.66(.52,13.39)
**Gravidity**				
Multigravida	35(11.11)	280(88.89)	1	
Primagravida	11(5.12)	204(94.88)	.431(.21,.86)	.87(.32, 2.31)
**Family Monthly income**				
< 2500 ETB	15(11.03)	121(88.97)	1	
2501 -5000ETB	17(9.77)	157(90.23)	.873(.42,1.81)	.43(.13,1.45)
5001- 8000ETB	7(7.37)	88(92.63)	.64(.25,1.63)	.39(.09,1.64)
> 8001ETB	7(5.60)	118(94.40)	.478(.18,1.22)	.19(.04,.87)
**Syphilis status**				
Yes	7(36.84)	12(63.16)	7.059(2.62,18.95)	10.28(2.80,37.62)*
No	39(7.63)	472(92.37)	1	

Keys: * = *P* < 0.05, *COR* Crude Odds Ratio, *AOR* Adjusted Odds Ratio, *CI* Confidence interval, 1 = reference category

## Discussion

HIV/AIDS has continued to be a significant public health problem throughout the globe. The proportion of HIV among pregnant women in the present study was found to be 8.68%. Among the three study sites a higher proportion was found in Felege Hiwot Comprehensive Specialized Hospital (10.40%), followed by University of Gondar Comprehensive Specialized Hospital (9.7%), and Debre Tabor Referral Hospital (4.6%).

The overall proportion of the present study was slightly lower than the two studies previously conducted in University of Gondar Referral Hospital (10.33%) [12] and (11.2%) [31]. Likewise, it was lower than a study done in the Yaoundé Central Hospital of Cameroon (13.1%) [32] and Abuja, Nigeria (11.5%) [33]. The difference between the current study and the two studies conducted in the University of Gondar Referral Hospital might be due to differences in the scope of the study setting in that the two studies were conducted in a single study site (referral hospital). The current study was rather done in three referral hospitals. One of the areas that the current study was conducted (Debre Tabor town) is quite different in terms of risk of acquiring the infection compared to the other two sites. Most of the nearby small urban areas surrounding Debre Tabor town are not identified as hotspot areas for HIV. On the other hand, most of the surrounding towns around Gondar and Bahir Dar cities are identified as hot spots for HIV. The other reason might be due to time difference. The previous two studies conducted in Gondar were before five years after which much has been done regarding HIV care and treatment such as the development and implementation of new policy and strategies as well as guidelines on combating HIV/AIDS and reaching the goal of ending HIV/AIDS by 2030 [23]. As a result, the findings from the previous studies were higher than the current study.

The discrepancy between the finding of the present study and those from Cameroon and Nigeria could be due to differences in the national prevalence of HIV as well as HIV prevalence among pregnant population of Ethiopia and these two countries. According to the 2014 Ethiopian national HIV Antenatal Care based Sentinel Surveillance; the prevalence of HIV among pregnant women attending ANC services was 2.2%. In Cameroon, on the other hand, the National Sentinel Surveillance of 2013 showed much higher prevalence (7.8%) of HIV among pregnant women compared to that of Ethiopia [11, 34]. Similarly, Nigeria ranks fourth globally with regard to HIV burden, which is the highest HIV burden in West and Central African sub-regions [35]. On the other hand, Ethiopia is one of the East African countries with a low (0.9%) national prevalence rate of HIV in 2017 [5].

Contrarily, the finding of the present study was higher than a study done in different parts of Ethiopia such as rural hospital of southern Ethiopia (0.2%) [36], western part of Ethiopia (1.0%) [37], the nationwide HIV Antenatal Care based Sentinel HIV Surveillance of Ethiopia (2.2%), Amhara region (6.1%) [11], Debre Berhan, Ethiopia (7.2%) [38], and Bahir Dar, Ethiopia (6.6%) [21]. Such a difference could be due to the socio-demographic characteristics of the study participants. Most of these studies were from the rural setting of Ethiopia that are known for having the lowest HIV prevalence rate in the country. The present study, on the other hand, was conducted in urban settings including cities identified as high-burden districts for HIV by the Ethiopian Ministry of Health [7, 11].

Similarly, the prevalence of HIV among pregnant women in the present study was found to be higher than a study done in Kumbo, Cameroon (4.9%) [39], Damaturu, Nigeria (4%) [40], Western Kenya (6.9%) [41], Guinea-Bissau 5.1% [6], Cuttack, India (0.5%) [42], Maharashtra, India (0.44%) [43], and the National Survey in Brazil (0.38%) [44]. Such a discrepancy might be due to differences in the socio-demographic characteristics, culture, prevention and control measures, risk factors and level of awareness about the disease. The other reason could be due to the increase in new HIV infection particularly among women [45]. Moreover, the proportion of HIV among pregnant women has been increasing recently since a significant number of women living with HIV have shown confidence in having children due to the accessibility and effectiveness of PMTCT care and treatment. In 2021, 81% of pregnant HIV-positive women worldwide had access to ART to prevent HIV transmission to their unborn children [1].

Regarding factors associated with HIV, completing secondary education, graduating from college or University, and having a family monthly income of greater than 8,001 ETB were variables found to be protective for maternal HIV sero status. Conversely, having a history of previous abortion, and being positive for syphilis were found to be a risk factor for maternal HIV sero status.

In the present study, women who completed secondary education as well as those who graduated from College/University had a less chance to be infected with HIV than women who were not able to read and write by 85% (AOR = 0.15; 95% CI: 0.04—0.53) and 97% (AOR = 0.03; 95% CI: 0.01—0.22), respectively. This finding is consistent with a study in Cape provinces of South Africa, demonstrating that every additional year of formal education reduces the risk of HIV infection by 10% [19]. In addition, a study in India showed that the prevalence of HIV was the highest among illiterate pregnant women [20]. This clearly shows that education plays a pivotal role in helping women to gain economic independence and having decision making power that eventually reduce social and economic vulnerability by mitigating risky sexual activities, increasing the ability to understand HIV and its prevention mechanisms. Furthermore, growing evidence shows that educated women have the power and capacity to be involved in the decision-making process of their life as well as their family’s lives. It was also demonstrated that more years of education are increasingly associated with safer sexual behavior and lower HIV prevalence [46].

Pregnant women with history of previous abortion had eight times greater odds to contract HIV infection than those who did not have such experiences (AOR = 7.73; 95% CI: 3.33—17.95). Similarly, the history of previous abortion was identified as an associated factor in a study done in Bahir Dar, Ethiopia with (AOR = 6.6; 95% CI: 2.50 -17.71) [21]. This could be due to the fact that the probability of acquiring HIV infection related with having unsafe previous abortions is high. On the other hand, previous studies showed that women living with HIV/AIDS have an increased risk of abortion. A study conducted in Nigeria revealed that women living with HIV infection were 1.4 times more likely to have spontaneous abortion compared to women living without the virus [47]. The reason could be women with HIV seropositive status are highly burdened with the multidimensional impact of HIV infection of either the physiological influence of the virus itself or the ART during pregnancy and its psychosocial pressure. The other reasons could be due to the fact that women living with HIV do not want to give birth losing hopes of getting an HIV free child; or due to unwanted pregnancies resulted from rape, incest, and failure in family planning [48, 49].

In the present study, women who were positive for syphilis were 10 times more likely to be infected with HIV than those women who were negative for syphilis (AOR = 10.28; 95% CI: 2.80—37.62). The reason might be due to the fact that these two infections have similar mode of transmission mainly unprotected sexual practice since both of them are sexually transmitted infections. The presence of syphilis infection can be a risk factor for HIV infection during unprotected sexual practice because the syphilis primary chance provides an easy entry point for HIV [50]. It is also apparent that HIV impairs the immune system which can make it easier for syphilis to develop [51]. It is also reported that syphilis is found to be associated with increased risk of HIV acquisition. A systematic review and meta-analysis study showed that the incidence of acquiring HIV among those exposed for syphilis were 2.67 times more likely than those who were not exposed [22]. Moreover, studies showed that HIV and syphilis have similar risk factors including risky sexual behaviors, consisting multiple sexual partners, unprotected sex with unknown persons, and sex under the influence of drugs or alcohol [32, 52].

Our study also showed that pregnant women who had a family monthly income of greater than 8001 ETB were less likely to be infected with HIV by 85% than those women who had monthly income of less than 2500 ETB. This finding is consistent with a study done in Nigeria where monthly income was independently associated with HIV infection demonstrating that the lower the monthly income the higher the risks of HIV infection [53]. This might be due to the fact that having a good socioeconomic status helps women not to put themselves in vulnerable circumstances that ultimately expose them to risky sexual behavior that can eventually lead them to becoming infected with HIV. Evidence also showed that HIV infection is highly related with social and economic status of a society disproportionately affecting individuals with lower social and economic conditions [53].

### Limitations of the study

This study has some limitations. One is that the study participants were pregnant women attending ANC. However, there are some pregnant women out there who could not access ANC services due to different reasons. Another limitation could be that our study did not include those pregnant women who had a follow up at other sectors of the health care tier since it was done at referral Hospitals only. This ultimately affects the reported figure which may underestimate or overestimate the proportion of HIV among the pregnant population. Still another limitation could be related with a wider confidence interval in the association between syphilis status and HIV status implying larger sample size was required.

### Conclusion and recommendation

This study revealed that the proportion of HIV among pregnant women was relatively high. Completion of secondary education, graduating from higher education institutions, relatively higher family monthly income, history of previous abortion, and positive syphilis status were significant predictors of maternal HIV sero status. Making formal education up to the higher level accessible for girls should be one of the top priorities of the government. Besides, the education curriculum should be strengthened to better address HIV education, educating women about HIV transmission methods and HIV prevention and control strategies using behavior change intervention strategy prepared for women to reduce their vulnerability; similarly, promoting family planning use to reduce unsafe abortion and syphilis, and screening and testing for syphilis should be strengthened. Finally, HIV screening and testing of pregnant women during ANC follow up and taking appropriate measures available is valuable to reduce the vertical and horizontal transmission of HIV infection which accounts for 90% of paediatrics HIV. This ultimately has a significant contribution in achieving the SDG-3 of ending HIV/AIDS and having HIV free generation by 2030. Finally, we would like to recommend researchers in this area to conduct studies with larger sample size by including pregnant women attending ANC services in the other health care tiers such as health centers in addition to referral Hospitals.

### Implication of the study

Although much has been done to end HIV by 2030, the finding suggests that HIV is still a significant public health issue in Ethiopia. It can also be deduced from the present study that almost all of the significant factors identified as risk and protective are modifiable and if appropriate actions could be done, one can achieve the SDG_3 calls for the eradication of HIV/AIDS and an HIV-free generation by 2030.

## Data Availability

The corresponding authors can share data when reasonable requests emerge.

## Abbreviations

AIDS: Acquired Immunodeficiency Syndrome
ETB: Ethiopian Birr; HIV: human immunodeficiency virus
ANC: Anti Natal Care
AOR: Adjusted Odds Ratio
ART: Antiretroviral Treatment
MTCT: Mother-to-child transmission
PMTCT: Prevention of mother-to-child transmission
MSSS: Maternity Social Support Scale
SDG: Sustainable Development Goals
SSA: Sub-Sahara Africa
UNAIDS: The Joint United Nations Programme on HIV/AIDS
UNFPA: The United Nations Population Fund
